# Post-Pandemic Resurgence of Pertussis in Southeastern Romania, 2024: Vaccination Gaps, Clinical Severity, and Regional Surveillance Performance

**DOI:** 10.3390/vaccines14070595

**Published:** 2026-07-04

**Authors:** Alina Plesea Condratovici, Mihaela Debita, Valerian Ionut Stoian, Catalin Plesea Condratovici, Ancuta Elena Tupu, Simona Steliana Tudor

**Affiliations:** Faculty of Medicine and Pharmacy, Medical-Pharmaceutical Research Center, “Dunarea de Jos” University of Galati, 800008 Galati, Romania; alina.plesea@ugal.ro (A.P.C.); catalin.plesea@ugal.ro (C.P.C.); anca.tupu@ugal.ro (A.E.T.); steliana.tudor@ugal.ro (S.S.T.)

**Keywords:** pertussis, whooping cough, *Bordetella pertussis*, epidemiological surveillance, vaccination coverage, immunity debt, pneumonia, post-pandemic resurgence, Romania, logistic regression

## Abstract

Background/Objectives: Following the COVID-19 pandemic, pertussis resurged sharply across Europe, with 209,674 cases reported in the EU/EEA in 2024. This study characterises the epidemiology of the 2024 pertussis resurgence across five counties of southeastern Romania, with emphasis on vaccination status, clinical severity, and regional surveillance performance. Methods: A retrospective, population-based analysis was conducted on 452 cases notified between February 2024 and January 2025, extracted from the national surveillance database. A pre-specified reclassification of PCR-positive cases yielded 326 confirmed cases. Categorical, non-parametric, correlation, and multivariate logistic regression analyses were performed. Results: The epidemic peaked in September 2024, with 56.0% of cases occurring between August and October. Children under five years accounted for 63.2% of confirmed cases, and 72.1% were not vaccinated according to age-appropriate schedule, predominantly due to parental refusal (43.0%) and non-attendance (36.6%). Pneumonia affected 36.8% of confirmed cases, ranging from 81.0% in infants under two months to 0% in adolescents. Age-appropriate vaccination was independently protective against pneumonia (adjusted OR = 0.53, 95% CI 0.29 to 0.96, *p* = 0.035; population attributable risk 37.3%). Significant inter-county heterogeneity was identified in PCR implementation (72 to 100%) and reporting delays. Conclusions: Vaccination gaps were the principal modifiable driver of the resurgence, supporting targeted coverage improvement and the introduction of a national maternal Tdap programme.

## 1. Introduction

Pertussis (whooping cough) is a highly contagious acute respiratory tract infection caused by *Bordetella pertussis* that has persisted as a recurring public health challenge throughout the era of mass vaccination. Although the implementation of childhood immunisation programmes from the mid-twentieth century onwards substantially reduced the global burden of disease, pertussis has neither been eradicated nor reduced to negligible endemic levels. The disease exhibits cyclical epidemic recurrences every three to five years, even in populations with high vaccination coverage, reflecting the interplay between waning vaccine-induced immunity, pathogen adaptation, and the progressive accumulation of susceptible individuals [1]. Following an unprecedented suppression of transmission during the COVID-19 pandemic, attributable to the implementation of non-pharmaceutical interventions that incidentally restricted respiratory pathogen circulation, notifications of pertussis began increasing sharply across the European Union and European Economic Area (EU/EEA) from 2023 onwards [2].

**Figure 1:**
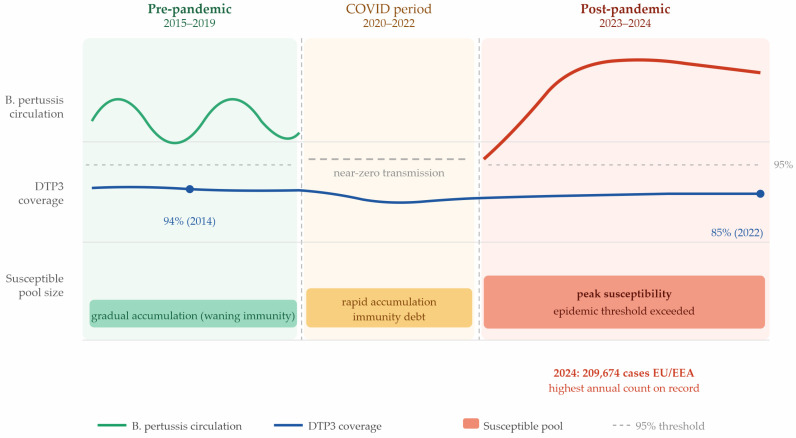
The immunity debt mechanism driving post-pandemic pertussis resurgence [4].

The scale of the post-pandemic resurgence has been substantial. Data compiled from international surveillance networks confirm a near-global pattern of epidemic rebound, with outbreaks reported across countries utilising both whole-cell and acellular pertussis vaccine formulations [3]. In the EU/EEA alone, 209,674 pertussis cases were reported by 29 countries in 2024, representing the highest annual notification count in the modern surveillance era, with infants under one year of age and adolescents aged 10 to 14 years recording notification rates of 318.5 and 204.2 per 100,000 population, respectively [4]. By June 2025, epidemic activity had been documented in at least 42 countries across multiple income strata, underscoring the global rather than regionally confined nature of the phenomenon [5]. The conceptual framework underlying this resurgence is illustrated in Figure 1, which depicts the concurrent processes of suppressed natural immunity boosting, declining vaccination coverage, and accelerating susceptible cohort accumulation that converged to generate the conditions for an unusually large epidemic cycle.

The schematic illustrates three concurrent processes across three epidemiological phases: suppression of *B. pertussis* circulation during 2020–2022 due to COVID-19 non-pharmaceutical interventions; progressive decline in DTP3 vaccination coverage in Romania from 94% in 2014 to 85% in 2022, persistently below the 95% herd-immunity threshold; and cumulative accumulation of susceptible individuals through waning vaccine-induced immunity and pandemic-associated immunisation disruption. Together, these processes created the conditions for the 2024 post-pandemic resurgence, during which 209,674 pertussis cases were reported across the EU/EEA [4].

The declining trend in vaccination coverage represents a structural vulnerability that extends beyond pertussis. According to the WHO position paper on pertussis vaccines, global DTP3 coverage has remained below the level required for sustained herd immunity in a substantial proportion of countries, and the post-pandemic period has further widened these gaps [6]. In Romania, pertussis surveillance is governed by the national methodology developed by the National Institute of Public Health, updated in January 2024, which defines standardised case classification criteria, notification procedures, and laboratory investigation protocols applicable across all county public health directorates [7]. The five counties of southeastern Romania under investigation encompass a combined population of approximately 2.37 million residents according to the 2021 national census [8]. The post-pandemic epidemiological context in Romania is characterised not only by pertussis resurgence but by a broader pattern of reemergence across vaccine-preventable diseases. Studies conducted in the same national surveillance framework have documented the positive impact of vaccination status on measles disease burden during the 2020 to 2024 epidemic period [9], identified complex spatio-temporal dynamics underlying the prolonged measles outbreak in Romania [10], and characterised clustering patterns in measles transmission that have direct methodological relevance to the analysis of vaccine-preventable disease outbreaks [11].

Against this background, no peer-reviewed study has characterised the epidemiology of the 2024 pertussis resurgence at subnational level in Romania, representing a substantive gap in the evidence required to guide targeted public health interventions. The present study addresses this gap by reporting the epidemiological characteristics of pertussis cases notified across five counties of southeastern Romania during the 2024 epidemic year. Specific objectives were: (i) to describe the temporal, geographic, and demographic distribution of notified cases; (ii) to characterise clinical presentation and complication profiles, with emphasis on vaccination status as a determinant of disease severity; (iii) to identify independent predictors of confirmed diagnosis and pneumonia through multivariate logistic regression; (iv) to evaluate key surveillance performance indicators across counties, including reporting delays and laboratory investigation rates; and (v) to contextualise the regional resurgence within the broader European and national epidemiological framework.

## 2. Theoretical Background

### 2.1. Etiology and Pathogenesis

*Bordetella pertussis* is a small, non-motile, aerobic, Gram-negative coccobacillus with exclusive human tropism, colonising the ciliated epithelium of the nasopharynx and upper respiratory tract through a regulated ensemble of adhesins and toxins whose expression is controlled by the BvgAS two-component signal transduction system. The organism’s pathogenicity is multifactorial and depends on the coordinated activity of filamentous hemagglutinin (FHA), fimbriae, pertactin (PRN), pertussis toxin (PT), adenylate cyclase toxin (ACT/CyaA), dermonecrotic toxin, and tracheal cytotoxin, each contributing to bacterial adhesion, immune evasion, and tissue damage [12]. Pertussis toxin, the principal immunodominant antigen incorporated in all acellular vaccine formulations, functions as an ADP-ribosylating exotoxin that inhibits G protein-coupled receptor signalling, induces lymphocytosis, and attenuates innate immune responses; adenylate cyclase toxin penetrates phagocytic cells to generate supraphysiological cAMP concentrations, paralysing bactericidal functions including oxidative burst and phagocytosis; and tracheal cytotoxin selectively destroys ciliated epithelial cells, impairing mucociliary clearance and sustaining nasopharyngeal colonisation [12].

A central challenge in contemporary pertussis control is the progressive adaptation of circulating *B. pertussis* strains to the selective pressure exerted by acellular vaccine formulations. Genomic analyses have documented antigenic divergence from vaccine reference strains in pertactin, FHA, and PT, with pertactin-deficient isolates now constituting up to 80% of clinical isolates in certain European countries, potentially reducing the effectiveness of acellular vaccines whose pertactin content was a key differentiating antigen from whole-cell formulations [13]. The immunological profile induced by acellular vaccination is characterised by predominantly Th2-skewed humoral responses, in contrast to the broader Th1/Th17 cellular immunity elicited by whole-cell vaccines or natural infection. The Th2-dominant polarisation associated with repeated antigen exposure, with relative deficiency of tissue-resident memory T-cell responses, represents a mechanistic parallel observed across distinct immunologically mediated conditions characterised by CD4+ T-cell subset imbalance [14]. In the context of pertussis, this polarisation results in insufficient protection against nasopharyngeal colonisation despite seroprotective antibody levels, enabling vaccinated individuals to sustain *B. pertussis* transmission in the absence of recognisable disease.

### 2.2. Clinical Spectrum

Classic pertussis in non-immune children progresses through three clinically distinguishable stages. The catarrhal stage, lasting one to two weeks, presents as an indistinguishable upper respiratory tract infection with coryza, low-grade or absent fever, and mild cough; this phase corresponds to peak nasopharyngeal bacterial load and highest infectivity. The paroxysmal stage, lasting two to eight weeks, is defined by repetitive forceful coughing episodes comprising up to 20 to 30 consecutive coughs in a single expiration, terminated by a forced inhalation producing the characteristic inspiratory whoop, frequently accompanied by post-tussive emesis, facial cyanosis, and periorbital petechiae. Fever is conspicuously absent in the paroxysmal stage, a diagnostically useful distinguishing feature. The convalescent stage is characterised by gradual reduction in paroxysm frequency and severity over weeks to months, with residual cough potentially persisting for up to three months, the basis for the historical designation “hundred-day cough” [15].

Age-dependent clinical variability has fundamental implications for disease recognition and surveillance. Neonates and very young infants frequently present without the classic inspiratory whoop, manifesting instead with apnoeic spells, choking episodes, bradycardia, and cyanosis that may not prompt immediate consideration of pertussis. Extreme leucocytosis, occasionally exceeding 100 × 10^9^/L, is a recognised marker of severe disease in this age group and is directly associated with pertussis-associated pulmonary hypertension, which carries a case fatality rate exceeding 70% despite aggressive cardiopulmonary support. Adolescents and adults, who constitute the primary community reservoir of transmission to unprotected infants, typically present with protracted non-specific cough illness exceeding two weeks, without the classic whoop, frequently misattributed to bronchitis or post-viral cough [15]. Critically, vaccination against pertussis does not prevent infection but substantially modifies clinical severity: vaccinated breakthrough cases are less likely to present with the classic inspiratory whoop, less likely to develop post-tussive emesis, and may complete the infectious period without seeking medical attention [16]. This phenomenon of attenuated, clinically silent transmission in vaccinated individuals is considered a primary driver of *B. pertussis* persistence in highly vaccinated populations and a fundamental limitation of acellular vaccine-based control strategies [16]. The three-month duration of potential infectiousness in untreated cases further amplifies community-level transmission [17].

### 2.3. Vaccination Strategies

Two fundamentally different pertussis vaccine categories have been deployed globally. Whole-cell vaccines (wP), comprising killed *B. pertussis* organisms, demonstrated efficacy of 80 to 95% in early controlled trials and induced broad humoral and cellular Th1/Th17 immune responses against multiple bacterial antigens. Despite their effectiveness, concerns regarding reactogenicity prompted progressive replacement by acellular formulations (aP) in high-income countries from the 1990s onwards [18]. Acellular vaccines contain two to five purified antigens and demonstrate substantially improved tolerability profiles; however, aP-induced immunity wanes markedly more rapidly, with serological markers declining to pre-vaccination levels within three to five years of primary series completion, and protection against pertussis decreases by approximately 30 to 40% per year following adolescent booster doses [19]. This differential durability between wP and aP protection is regarded as a structural contributor to pertussis resurgence in high-income countries that transitioned to acellular formulations.

Maternal vaccination with Tdap during the third trimester of pregnancy (27 to 36 weeks gestation) represents the most effective currently available strategy for protecting neonates and very young infants through transplacental transfer of maternal IgG anti-PT antibodies. Observational and randomised trial evidence consistently reports Tdap maternal vaccination effectiveness of 80 to 93% against confirmed infant pertussis in the first months of life [20]. Given that the period before completion of the primary immunisation series constitutes the interval of highest risk for severe outcomes and death, antenatal immunisation programmes are a public health priority specifically for protecting the most vulnerable age group; delays in maternal vaccination or suboptimal antenatal programme coverage are directly associated with increased infant morbidity [21]. Protecting neonates through maternal immunisation is particularly critical in the context of the 2024 resurgence, during which the diagnostic and clinical challenges of pertussis in early infancy have been extensively documented [22]. Global immunisation strategies, including the WHO Immunization Agenda 2030, emphasise that achieving and sustaining vaccination coverage above 95% across all birth cohorts, combined with targeted strategies for high-risk populations including pregnant women, is essential to interrupt pertussis transmission cycles [23].

In Romania, the national immunisation calendar prescribes a primary pertussis series with hexavalent vaccine (DTaP-IPV-Hib-HepB) at two and eleven months of age, followed by a booster dose with tetravalent vaccine (DTaP-IPV) at five years, in accordance with WHO guidance for high-coverage settings [7]. A national maternal Tdap vaccination programme has not been established, representing a structural gap in the protection of the youngest and most vulnerable infants.

### 2.4. Diagnostic Approach

Laboratory diagnosis of pertussis relies on three methodological approaches whose diagnostic yields diverge markedly according to the interval between symptom onset and specimen collection, with important consequences for the completeness and accuracy of passive surveillance data.

**Figure 2:**
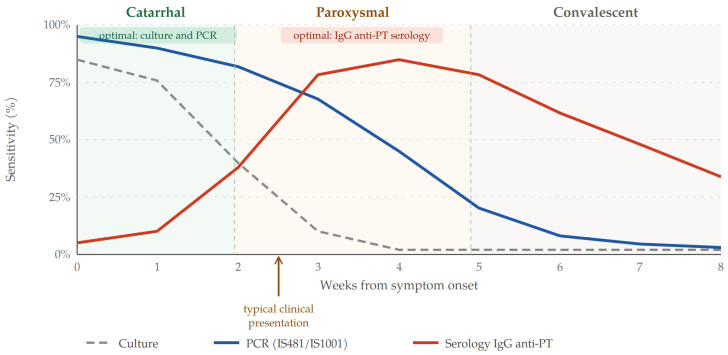
Sensitivity of pertussis diagnostic methods by time from symptom onset.

Real-time PCR targeting insertion sequences IS481 and IS1001 of *B. pertussis* has become the preferred first-line diagnostic method across EU/EEA reference laboratories, offering sensitivity of 80 to 99% during the first three weeks of illness and turnaround times of 24 to 48 h [24]. PCR can be performed on nasopharyngeal specimens collected even after antibiotic initiation and retains positivity for three to four weeks from symptom onset. Culture of *B. pertussis* on selective media (Bordet-Gengou or Regan-Lowe agar) retains 100% specificity and enables strain characterisation and antimicrobial susceptibility testing; however, its sensitivity declines sharply from approximately 85% in the first week to below 20% after the third week of illness and approaches zero following antibiotic administration, limiting its practical utility in routine surveillance settings. Serological diagnosis based on the detection of serum IgG antibodies against pertussis toxin (IgG anti-PT) by ELISA using WHO-standardised reference sera is the method of choice for patients presenting in the paroxysmal or convalescent stages. A single serum IgG anti-PT titre of 100 IU/mL or above, in the absence of a pertussis-containing vaccine administered within the preceding 12 months, is interpreted as indicative of recent infection [25]. This threshold, validated against paired sera criteria, demonstrates specificity approaching 100% for *B. pertussis* infection given the unique production of PT by this species [26]. The time-dependent diagnostic utility of culture, PCR, and serology is summarised in Figure 2.

The figure shows how the diagnostic yield of culture, PCR, and IgG anti-PT serology varies according to the interval from symptom onset and across the catarrhal, paroxysmal, and convalescent stages. Culture has the highest sensitivity early in disease, PCR remains useful during the first three to four weeks, and serology becomes most informative during the later paroxysmal and convalescent phases. Adapted from ECDC guidance [24,25] and de Melker et al. [26].

The practical consequence of this temporal mismatch between optimal diagnostic windows and typical healthcare-seeking behaviour is a systematic downward bias in laboratory-confirmed case counts relative to true incidence. In passive surveillance systems, the probability of obtaining a positive laboratory result is directly dependent on the delay between symptom onset and specimen collection, which in turn depends on clinician recognition and healthcare access. This diagnostic gap disproportionately affects older patients, vaccinated individuals with attenuated presentations, and populations with limited primary care access, and is a primary determinant of the under-notification that characterises pertussis surveillance in all existing passive reporting systems, including the Romanian national system [27].

### 2.5. Global and European Epidemiological Trends

Pertussis has historically followed cyclical epidemic recurrences every three to five years in the European region, even in populations with sustained high vaccination coverage. During the pre-pandemic decade (2010 to 2019), EU/EEA countries reported major epidemic peaks in 2012, 2016, and 2019, with notification rates ranging between 5 and 11 per 100,000 population and substantial inter-country heterogeneity reflecting differences in surveillance sensitivity, laboratory capacity, and vaccine schedules [28]. Throughout this period, two divergent patterns emerged across Europe: in countries with sustained high infant coverage, the age distribution of disease progressively shifted toward adolescents and adults with waned immunity, while in countries with lower or disrupted coverage, infant disease and hospitalisation remained predominant. The global disease burden of pertussis among adults has been quantified using Global Burden of Disease (GBD) 2021 data, which documented progressive increases in adult incidence in multiple lower-income regions between 2009 and 2019 despite declining rates in high-income countries, underscoring persistent structural inequities in pertussis control [29]. Among individuals under 20 years of age, global pertussis incidence declined by 77.7% between 1990 and 2021, though this aggregate improvement conceals marked regional disparities, with low sociodemographic index regions continuing to bear the greatest burden of infant deaths and disability-adjusted life years [30].

The COVID-19 pandemic introduced an acute perturbation into this established epidemiological cycle. Non-pharmaceutical interventions implemented between 2020 and 2022 suppressed *B. pertussis* circulation by over 90% across most EU/EEA countries relative to pre-pandemic levels. Simultaneously, pandemic-associated disruptions to primary healthcare infrastructure precipitated declines in DTP3 coverage in multiple countries. The convergence of these processes generated the immunological preconditions for an unusually large post-pandemic epidemic, which materialised as the 2023 to 2024 resurgence described in Section 1.

### 2.6. Pertussis Surveillance in Romania

Pertussis is a legally notifiable communicable disease in Romania subject to mandatory reporting under Government Decision HG no. 657/2022 [31], which defines the national notifiable diseases list and applicable reporting timelines. The national pertussis surveillance methodology, updated by the National Institute of Public Health in January 2024 [7], defines standardised case classification criteria aligned with EU/ECDC definitions: possible (clinical criteria only), probable (clinical plus epidemiological link to a confirmed case), confirmed (clinical plus laboratory criteria), and infirmed (negative laboratory investigation). Electronic transmission of case databases in EpiInfo format from county public health directorates (DSPs) to regional public health centres (CRSPs) on a weekly basis, and to the National Centre for Surveillance and Control of Communicable Diseases (CNSCBT) via CRSPs, constitutes the operational backbone of the national system. Romania reports pertussis data to ECDC through the TESSy electronic surveillance platform.

The evolution of DTP3 vaccination coverage in Romania over the preceding decade reflects a convergence of structural healthcare system challenges, supply chain disruptions, and growing vaccine hesitancy. WHO/UNICEF estimates document a sustained decline from 94% in 2014 to 85% in 2022, with particularly sharp drops coinciding with documented vaccine supply shortages [6]. At 85%, national coverage falls ten percentage points below the 95% threshold considered necessary to prevent sustained community transmission, and substantial subnational disparities in coverage between counties and between urban and rural areas create localised pockets of heightened epidemic vulnerability even when national averages appear acceptable.

## 3. Materials and Methods

### 3.1. Study Design and Setting

A retrospective, population-based, observational epidemiological study was conducted using routine passive surveillance data. The study encompassed five counties of Romania’s South-East development region (NUTS-2 RO22): Braila (BR), Buzau (BZ), Constanta (CT), Galati (GL), and Tulcea (TL). This region borders the Danube Delta and the Black Sea coastline and includes both urban industrial centres and predominantly rural agricultural areas. The combined resident population of the five counties was approximately 2.37 million at the 2021 national census [8]. The study period included all pertussis cases with recorded symptom onset between 1 February 2024 and 31 January 2025, capturing the full epidemic wave from its initiation through return to baseline notification rates.

### 3.2. Data Source and Study Population

Surveillance data were extracted from the national EpiInfo pertussis database maintained by the County Public Health Directorates (DSPs) of the five study counties, populated through the standardised Pertussis Case Surveillance Form (*Fisa de supraveghere a cazului de tuse convulsiva*) in accordance with the INSP pertussis surveillance methodology updated 9 January 2024 [7]. This form is completed for every case notified to the DSP as suspected, probable, or confirmed pertussis under the mandatory reporting provisions of HG 657/2022 [31]. Data were transmitted to the study team by the National Institute of Public Health, Centre for Surveillance and Control of Communicable Diseases (INSP-CNSCBT), following an official request, with the stipulation that data be used exclusively with attribution to the source of provenance. Data extraction encompassed all records with onset dates falling within the pre-specified study period, irrespective of final case classification. One record containing an unspecified county code was excluded from geographic and county-stratified analyses and retained only in aggregate descriptive statistics. No further exclusion criteria were applied. The final analytical cohort comprised 452 reported cases, of which 451 had complete county-level data available.

### 3.3. Case Definitions and Reclassification

Cases were classified according to the EU/ECDC-aligned definitions adopted in the INSP 2024 national surveillance methodology [7]. A possible case is any person presenting with cough lasting a minimum of two weeks combined with at least one of the following: paroxysmal coughing episodes, inspiratory whoop, or post-tussive vomiting; or any person diagnosed with pertussis by an attending physician; or apnoeic episodes in an infant under one year of age. A probable case meets clinical criteria and has a documented epidemiological link with a laboratory-confirmed case. A confirmed case meets both clinical and laboratory criteria, with laboratory confirmation defined as at least one of the following: isolation of *B. pertussis* in culture; detection of *B. pertussis* nucleic acid by PCR in a nasopharyngeal specimen; or serum IgG anti-PT of 100 IU/mL or above by ELISA in the absence of a pertussis-containing vaccine dose within the preceding 12 months. An infirmed case is one in which laboratory investigations yield negative results.

**Figure 3:**
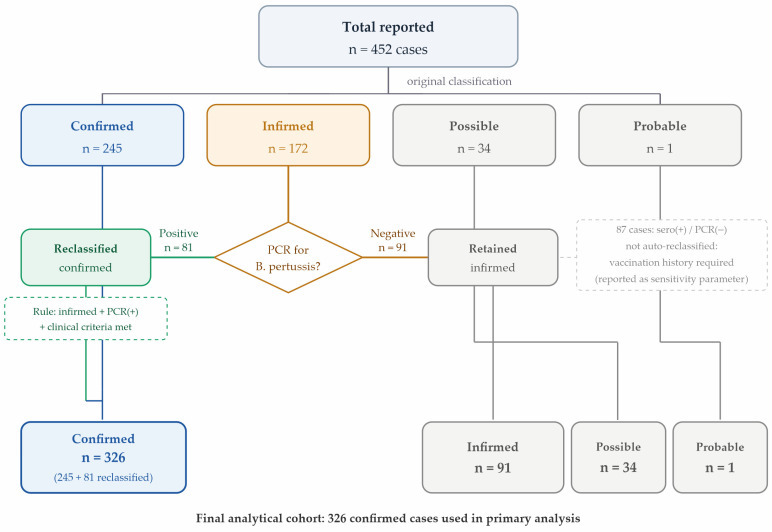
Case reclassification flowchart and final analytical cohort.

Cross-tabulation of the original database classifications against documented laboratory results revealed a systematic inconsistency: 81 of 83 cases (97.6%) with a positive PCR result for *B. pertussis* carried an original classification of “infirmed”, whereas 96.7% of cases originally classified as “confirmed” lacked any positive laboratory result. This pattern is directly inconsistent with the INSP 2024 case definition and is most consistent with classifications having been finalised prior to receipt of laboratory results without subsequent record update, a recognised limitation of sequential passive surveillance data entry workflows [7]. A single pre-specified reclassification rule was applied before all analyses: any case originally classified as “infirmed” with a documented positive PCR result for *B. pertussis* and concurrent clinical criteria met was reclassified as “confirmed”. This rule was verified to apply to all 81 affected records. Cases with positive serology but negative or absent PCR originally classified as “infirmed” (n = 87) were not subjected to automatic reclassification, as serological titres require individual assessment of vaccination history within the preceding 12 months before infection can be distinguished from vaccine-induced response [26]. These 87 cases are reported descriptively as a sensitivity parameter. Following reclassification, the study cohort comprised 326 confirmed cases, 91 infirmed cases, 34 possible cases, and 1 probable case, as illustrated in Figure 3.

The flowchart summarises the PCR-based reclassification procedure applied to the pertussis surveillance database of 452 reported cases from southeastern Romania in 2024. Cases originally classified as “infirmed” were assessed for PCR positivity, and 81 records with a documented positive PCR result for *B. pertussis* and compatible clinical criteria were reclassified as confirmed, in accordance with the INSP 2024 case definition [7]. Cases with positive serology but absent or negative PCR (n = 87) were not reclassified automatically, because IgG anti-PT titres above 100 IU/mL require individual verification of recent vaccination history to distinguish infection from vaccine-induced response [26]. The final analytical cohort comprised 326 confirmed, 91 infirmed, 34 possible, and 1 probable case.

Following reclassification, the primary analysis was conducted on the 326 confirmed cases. The complete cohort of 452 reported cases was used for analyses of surveillance system performance, which require the full notification dataset irrespective of classification outcome.

### 3.4. Variables and Data Collection

The following variable categories were extracted from the standardised surveillance form. Demographic variables included age in completed years and age in months for children under one year, sex, county of residence, locality, and residential setting (urban or rural). Temporal variables included date of symptom onset, date of isolation, date of DSP notification, and date of final case classification; reporting delay was defined as the interval in days between symptom onset and DSP notification. Clinical variables comprised the five case definition criteria (cough lasting at least two weeks, paroxysmal cough, inspiratory whoop, post-tussive vomiting, apnoea in infants under one year) and three complication variables (pneumonia, acute encephalopathy, convulsions), each coded as Yes, No, or missing. Vaccination variables included total number of prior DTPa doses received before diagnosis, age-appropriate vaccination status (Yes or No), reason for non-vaccination (coded as: 0, medical contraindication; 1, parental refusal; 2, non-attendance at scheduled appointments; 3, prior confirmed pertussis; 4, other reason), and dates of each recorded dose. Age-appropriate vaccination status was defined exclusively against the paediatric national immunisation schedule (primary series by 11 months and a booster at five years). For individuals aged 18 years or above, age-appropriate status was classified as not applicable, because the Romanian immunisation programme does not include a routine adult pertussis booster and no standardised adult schedule exists against which currency of vaccination could be determined. For individuals aged 18 years or above, age-appropriate vaccination status was classified as not applicable, because the Romanian immunisation programme does not include a routine adult pertussis booster and no standardised adult schedule exists against which vaccination currency could be determined. In descriptive age-stratified tables, adults were therefore reported as “N/A” for the age-appropriate vaccination variable. In multivariable models, the primary analysis was retained on the full analytical cohort to preserve comparability with the main case-definition and severity analyses; to address the non-applicability of the age-appropriate vaccination variable in adults, both models were repeated after excluding individuals aged 18 years or above as a pre-specified sensitivity analysis. Laboratory variables comprised culture result, PCR on nasopharyngeal specimen (performed Yes or No; result coded as 0 negative, 1 positive, 2 indeterminate), first serum IgG anti-PT (date and quantitative result in IU/mL with categorical interpretation: 0 negative below 40 IU/mL, 1 positive at or above 100 IU/mL, 2 equivocal 40 to 99 IU/mL), second serum, and final serology result.

Age groups were defined a priori for descriptive analyses as: below 2 months, 2 to 5 months, 6 to 11 months, 1 to 4 years, 5 to 9 years, 10 to 14 years, 15 to 17 years, and 18 years or above. For regression modelling, age was treated as a five-level ordinal variable (0, below 1 year; 1, 1 to 4 years; 2, 5 to 9 years; 3, 10 to 17 years; 4, 18 years or above).

### 3.5. Statistical Analysis

Statistical analyses were performed using IBM SPSS Statistics version 29.0 (IBM Corp., Armonk, NY, USA). Categorical variables were summarised as frequencies and percentages, and continuous variables as mean with standard deviation (SD) or median with interquartile range (IQR), according to their distribution. The normality of continuous variables was tested using the Shapiro–Wilk test. Both age (W = 0.625, *p* < 0.001) and reporting delay (W = 0.883, *p* < 0.001) departed significantly from a normal distribution, and non-parametric tests were therefore used for these variables. A two-tailed *p*-value below 0.05 was considered statistically significant.

Associations between categorical variables were assessed using the Pearson chi-square test, with Fisher’s exact test applied when any expected cell count was below five. Continuous variables were compared between two groups using the Mann–Whitney U test, and across more than two groups using the Kruskal–Wallis test; where the Kruskal–Wallis test was significant, pairwise comparisons were performed with the Mann–Whitney U test and Bonferroni correction for multiple testing. Correlations between continuous and ordinal variables were evaluated using the Spearman rank correlation coefficient.

The analytical approach drew on multivariable modelling strategies previously applied to clinical and population health datasets within the same research group, including cardiovascular risk assessment [32], AI-assisted screening in athlete populations [33], machine learning-based health assessment in young populations [34], and predictive modelling of multivariable systems [35].

Two binary logistic regression models were built using the enter method. Model A assessed factors associated with confirmed diagnosis (confirmed versus non-confirmed) in the full cohort (n = 452), and Model B assessed factors associated with pneumonia among confirmed cases (n = 326). Both models included sex, area of residence, age-appropriate vaccination status, and age group as covariates. Results are presented as adjusted odds ratios (OR) with 95% confidence intervals (CI). Model fit was evaluated using the Hosmer–Lemeshow goodness-of-fit test [36] and the Nagelkerke R-squared [37]. Model A was statistically significant (omnibus chi-square = 16.95, *p* = 0.002; Nagelkerke R^2^ = 0.053), as was Model B (omnibus chi-square = 54.95, *p* < 0.001; Nagelkerke R^2^ = 0.212; Hosmer–Lemeshow *p* = 0.352).

The population attributable risk (PAR) of non-vaccination for pneumonia was calculated using the Levin formula [38], based on the proportion of unvaccinated individuals among confirmed cases and the relative risk of pneumonia in unvaccinated compared with vaccinated cases, with the 95% confidence interval estimated by bootstrap resampling (10,000 replications). An overall crude incidence rate per 100,000 population was calculated for the five-county study region using the total domicile population recorded at the 2021 Population and Housing Census [8], which constituted the most recent official population denominator accessible to the authors for the full region. County-specific and age-specific incidence rates were not computed, as disaggregated resident-population denominators by county and age band for the 2024 epidemic year were not available to the authors at the time of analysis; the overall crude rate is therefore reported as an approximate measure intended to support comparison with other European jurisdictions rather than to provide a definitive year-specific estimate.

### 3.6. Ethical Considerations

The study was conducted exclusively using anonymised routine communicable disease surveillance data transmitted by the National Institute of Public Health (INSP-CNSCBT) following an official institutional request, under the mandatory reporting provisions of HG 657/2022 [31], which constitutes a legal basis for data processing pursuant to Article 9(2)(i) of Regulation (EU) 2016/679 [39]. The surveillance database contains no direct patient identifiers; each record is coded using initials, date of birth, and a unique epidemiological reference number assigned by the notifying DSP. Data processing complied with Romanian Law no. 190/2018 on data protection measures supplementary to the GDPR. In accordance with applicable national regulations and European guidance on observational studies using existing public health surveillance data, individual ethics committee review was not required [40]. Authors should confirm this determination with their institutional review board prior to submission.

## 4. Results

### 4.1. Overview of the Study Cohort

A total of 452 pertussis cases were reported across the five study counties between 1 February 2024 and 31 January 2025. Based on the combined 2021 census domicile population of approximately 2.37 million for the five study counties [8], this corresponds to an overall crude incidence of 19.1 per 100,000 population for all reported cases and 13.8 per 100,000 for laboratory-confirmed cases during the study period. Following the pre-specified PCR-based reclassification procedure, the cohort comprised 326 confirmed cases (72.1%), 91 infirmed cases (20.1%), 34 possible cases (7.5%), and 1 probable case (0.2%). Among confirmed cases, 173 were female (53.1%) and 153 were male (46.9%). The mean age of the total cohort was 6.1 years (standard deviation 9.7), with a median of 3 years (interquartile range [IQR] 0 to 7; range 0 to 57 years). Both age and reporting delay demonstrated significantly non-normal distributions (Shapiro–Wilk W = 0.625 and W = 0.883, respectively; both *p* below 0.001), confirming the appropriateness of non-parametric methods. General cohort characteristics are summarised in Table 1.

### 4.2. Temporal Distribution

**Figure 4:**
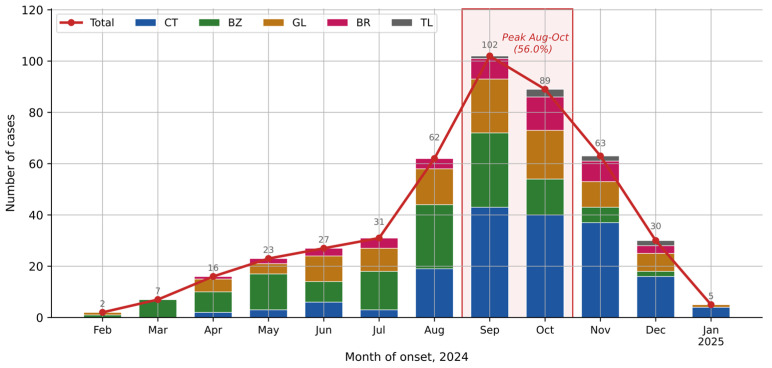
Monthly epidemic curve of pertussis cases by county, southeastern Romania, February 2024 to January 2025.

Case notifications were recorded in all months of the study period, with a markedly asymmetric seasonal distribution (Figure 4). Monthly counts increased progressively from February 2024 (n = 2) through the summer months, reaching a peak of 102 cases in September 2024. The three-month period from August through October 2024 accounted for 253 cases, representing 56.0% of the annual total and constituting the epidemic peak. A secondary wave of lower intensity was observed from November through December 2024 (n = 91 combined), followed by a sharp decline to 2 cases in January 2025, indicating resolution of the epidemic wave within the study period. The temporal distribution was broadly consistent across all five counties, with Constanta and Buzau contributing the largest absolute case counts throughout the peak period.

The monthly distribution shows a progressive increase in reported cases from February 2024, a main epidemic peak in September 2024, and a sharp decline by January 2025. Constanta and Buzau contributed the largest absolute numbers of cases during the peak period.

### 4.3. Geographic Distribution

The distribution of reported cases across the five counties was heterogeneous (Table 2). Constanta recorded the highest absolute case count (n = 173; 38.4%), followed by Buzau (n = 129; 28.6%), Galati (n = 91; 20.2%), Braila (n = 50; 11.1%), and Tulcea (n = 8; 1.8%). After reclassification, county-specific confirmation rates ranged from 52.7% in Buzau to 96.0% in Braila, with the inter-county difference being highly significant (chi-square = 53.43, df = 4, *p* below 0.001). Rural residential setting was significantly associated with lower odds of confirmed classification across the full cohort (chi-square = 7.43, *p* = 0.006).

### 4.4. Demographic Profile

**Figure 5:**
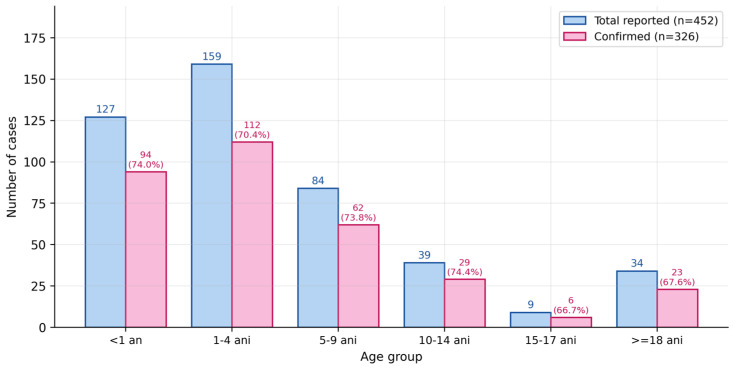
Age distribution of total reported and confirmed pertussis cases.

Children under five years of age constituted the dominant affected group, accounting for 63.3% of all reported cases (n = 286) and 63.2% of confirmed cases (n = 206; Figure 5). The largest single age group was 1 to 4 years (35.2% of all cases; 34.4% of confirmed), followed by infants under one year (28.1% of all cases; 28.8% of confirmed). Among infants, those under two months of age (n = 25; 21 confirmed) represent the subgroup at highest clinical risk, as they are ineligible for the first vaccine dose at the time of illness onset. Adults aged 18 years or above accounted for 7.5% of reported cases (n = 34; 23 confirmed). Sex was not significantly associated with confirmed status (chi-square = 0.46, *p* = 0.500). Kruskal–Wallis analysis identified significant differences in age distribution across counties (H = 15.47, *p* = 0.004), reflecting a higher proportion of adult cases in Galati and a predominantly pediatric case distribution in Buzau.

The figure demonstrates that children under five years of age represented the dominant affected group among both total reported and confirmed cases. Infants under one year and children aged 1–4 years accounted for most confirmed cases, whereas adolescents and adults represented a smaller proportion of the cohort.

### 4.5. Clinical Presentation and Complications

Among the 326 confirmed cases, paroxysmal cough was the most frequently documented clinical feature, present in 268 of 326 cases (82.2%), followed by cough lasting at least two weeks in 196 of 323 evaluable cases (60.7%), post-tussive vomiting in 142 of 324 (43.8%), inspiratory whoop in 120 of 326 (36.8%), and apnoeic episodes in 29 of 291 evaluable infants (10.0%).

Pneumonia was the most common complication, occurring in 120 of 326 confirmed cases (36.8%). The pneumonia rate exhibited a strong and statistically significant inverse relationship with age (chi-square = 49.46, df = 7, *p* below 0.001; Spearman rho = −0.373, *p* below 0.001): 81.0% of confirmed cases in the below-2-month group developed pneumonia, declining progressively through subsequent age groups to 0% in the 15- to 17-year group and 8.7% in adults aged 18 years or above. Infants under one year had 4.13-fold higher odds of developing pneumonia compared to older patients (chi-square = 30.82, *p* below 0.001). Acute encephalopathy was documented in 2 confirmed cases (0.6%) and convulsions in 1 case (0.3%). No deaths were recorded in the confirmed case cohort. Pneumonia rates by age group and vaccination status are presented in Table 3.

### 4.6. Vaccination Status Analysis

**Figure 6:**
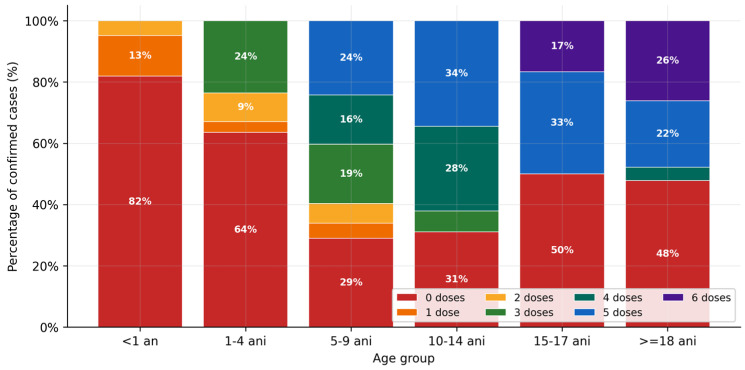
Vaccine dose distribution by age group in confirmed cases.

Among the 326 confirmed cases, 235 (72.1%) were not vaccinated according to the age-appropriate national schedule, while 91 (27.9%) had received the recommended number of doses for their age. The distribution of vaccination status differed significantly across age groups (chi-square = 22.12, df = 5, *p* below 0.001; Figure 6). In infants under one year, 83 of 94 confirmed cases (88.3%) were not age-appropriately vaccinated, including 21 (22.3%) aged below two months who had not yet reached the minimum eligible age for the first vaccine dose.

The figure shows that zero-dose cases predominated among infants and younger children, whereas older children and adolescents more frequently had received three or more pertussis-containing vaccine doses before diagnosis.

**Figure 7:**
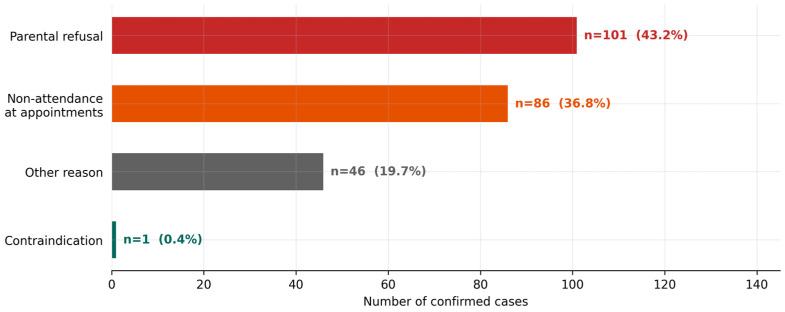
Reasons for non-vaccination among confirmed cases.

Among confirmed cases not vaccinated per age (n = 235), the leading documented reasons were parental refusal (n = 101; 43.0%) and non-attendance at scheduled vaccination appointments (n = 86; 36.6%), together accounting for 79.6% of non-vaccination. Other administrative or undocumented reasons accounted for 46 cases (19.6%), and a medical contraindication was recorded in 1 case (0.4%; Figure 7).

The figure shows that parental refusal and non-attendance at scheduled vaccination appointments were the leading documented reasons for failure to receive age-appropriate vaccination. Together, these two categories accounted for most non-vaccinated confirmed cases.

Among confirmed cases who had received at least one pertussis-containing dose (n = 140), the interval between the last recorded dose and symptom onset increased progressively with age, from a median of 20.9 months in children aged 1–4 years to 51.8 months at 5–9 years, 74.7 months at 10–14 years, and 203.4 months in adults aged 18 years or above. This interval showed a weak but statistically significant inverse correlation with pneumonia (Spearman rho = −0.203, *p* = 0.016): longer intervals since last vaccination were associated with lower pneumonia frequency, reflecting the concentration of severe disease in the youngest, recently- or not-yet-vaccinated infants rather than in older individuals with waned immunity. Among vaccinated cases aged 10 years or above (n = 34; median interval 102.2 months), only 4 (11.8%) developed pneumonia.

The distribution of doses received before diagnosis showed that 186 of 326 confirmed cases (57.1%) had received zero doses at the time of illness onset. Vaccination status was significantly associated with pneumonia occurrence: the crude odds ratio for pneumonia in unvaccinated compared with vaccinated confirmed cases was 2.43 (chi-square = 9.43, *p* = 0.002). The population attributable risk (PAR) for non-vaccination as a determinant of pneumonia was 37.3% (95% CI 15.8 to 58.5%; crude relative risk RR = 1.83; proportion unvaccinated = 72.1%), indicating that approximately one third of pneumonia cases in confirmed pertussis were attributable to the absence of age-appropriate vaccination.

### 4.7. Laboratory Diagnostics

Among the 326 confirmed cases, laboratory confirmation was available in 89 (27.3%): 82 by positive PCR for *B. pertussis*, 7 by positive IgG anti-PT serology (≥100 IU/mL in the absence of vaccination within the preceding 12 months), and 1 by positive culture. The remaining 237 cases (72.7%) were classified as confirmed on clinical grounds, in accordance with the national case definition, which permits confirmation based on a diagnosis of pertussis by the attending physician [7]. This reflects the predominantly clinical basis of case confirmation in routine passive surveillance rather than universal laboratory ascertainment, consistent with the diagnostic under-ascertainment inherent to passive pertussis surveillance systems.

PCR testing of nasopharyngeal specimens was performed in 290 of 326 confirmed cases (88.9%), of which 82 yielded positive results for *B. pertussis* (82 of 290; 28.3%). PCR testing rates varied significantly across counties (chi-square = 93.67, df = 4, *p* below 0.001): Braila, Galati, and Tulcea achieved near-complete testing rates (100%, 99%, and 100%, respectively), while Buzau demonstrated substantially lower implementation (72%). Serology (IgG anti-PT ELISA) was performed in only 50 of 326 confirmed cases (15.3%), of which 11 recorded positive titres at or above 100 IU/mL. Culture was performed in 2 cases across the entire cohort (0.4%), confirming the near-complete abandonment of this method in routine surveillance practice.

### 4.8. Multivariate Analysis

**Figure 8:**
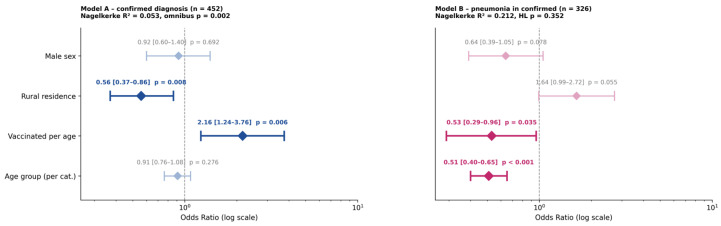
Forest plot of adjusted odds ratios for confirmed diagnosis and pneumonia.

Results from both logistic regression models are presented in Table 4 and visualised as a forest plot in Figure 8.

The forest plot presents adjusted odds ratios and 95% confidence intervals from the two multivariable logistic regression models. Age-appropriate vaccination was independently associated with lower odds of pneumonia, while younger age was associated with increased pneumonia risk among confirmed pertussis cases.

In Model A (outcome: confirmed diagnosis, n = 452), two covariates reached statistical significance. Rural residence was independently associated with lower odds of confirmed classification (adjusted OR = 0.56, 95% CI 0.37 to 0.86, *p* = 0.008). Age-appropriate vaccination was independently associated with higher odds of confirmed classification (adjusted OR = 2.16, 95% CI 1.24 to 3.76, *p* = 0.006). Sex (adjusted OR = 0.92, 95% CI 0.60 to 1.40, *p* = 0.692) and age group (adjusted OR = 0.91, 95% CI 0.76 to 1.08, *p* = 0.276) were not independently significant. The model was statistically significant overall (omnibus chi-square = 16.95, *p* = 0.002), with a Nagelkerke R^2^ of 0.053, indicating modest explanatory power as expected for a surveillance-based model.

**Figure 9:**
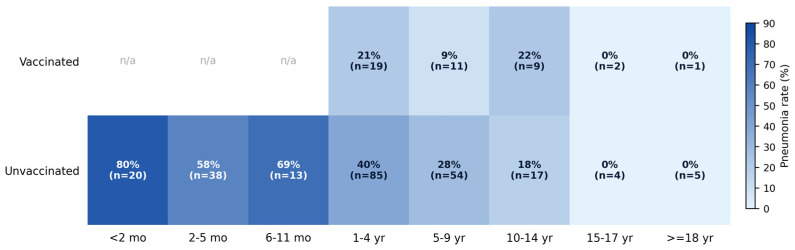
Pneumonia rate by age group and vaccination status among confirmed pertussis cases.

In Model B (outcome: pneumonia among confirmed cases, n = 326), two covariates reached the significance threshold and two showed borderline associations. Younger age group was the strongest independent predictor of pneumonia (adjusted OR = 0.51, 95% CI 0.40 to 0.65, *p* < 0.001), indicating that each successive age category was associated with approximately halved odds of pneumonia. Age-appropriate vaccination was independently protective against pneumonia (adjusted OR = 0.53, 95% CI 0.29 to 0.96, *p* = 0.035), corresponding to approximately half the odds of developing pneumonia among vaccinated compared with unvaccinated confirmed cases, after adjustment for age, sex, and area of residence. The age-specific gradient in pneumonia frequency by vaccination status is shown in Figure 9. Rural residence showed a borderline positive association with pneumonia (adjusted OR = 1.64, 95% CI 0.99 to 2.72, *p* = 0.055), and male sex a borderline inverse association (adjusted OR = 0.64, 95% CI 0.39 to 1.05, *p* = 0.078), though neither reached the significance threshold. The Hosmer–Lemeshow test indicated adequate model fit (chi-square = 8.89, df = 8, *p* = 0.352), and the Nagelkerke R^2^ was 0.212. Because age-appropriate vaccination status is defined only for the paediatric population, both multivariable models were repeated excluding adults aged 18 years or above as a sensitivity analysis. In Model A (confirmed diagnosis, n = 418), age-appropriate vaccination remained independently associated with confirmed status (adjusted OR = 2.01, 95% CI 1.13 to 3.56, *p* = 0.017), and in Model B (pneumonia among confirmed cases, n = 303), the protective effect of vaccination against pneumonia was essentially unchanged (adjusted OR = 0.50, 95% CI 0.27 to 0.91, *p* = 0.024). The exclusion of adults did not materially alter any of the principal associations, confirming the robustness of the findings to the handling of adult vaccination status.

A second sensitivity analysis assessed the influence of the PCR-based reclassification by repeating the principal analyses on the 245 originally confirmed cases only, excluding the 81 reclassified cases. The key findings were preserved: children under five years represented 68.6% of cases, 77.1% were not age-appropriately vaccinated, and pneumonia affected 40.4%. The protective effect of age-appropriate vaccination against pneumonia remained significant and of similar magnitude (adjusted OR = 0.44, 95% CI 0.21 to 0.92, *p* = 0.029, versus OR = 0.53 in the full reclassified cohort), confirming that the reclassification procedure did not materially alter the principal conclusions and, if anything, the association was marginally stronger in the unreclassified cohort.

The figure shows that pneumonia frequency was highest among infants, particularly those below two months of age, and declined progressively with increasing age. Across age groups, cases not vaccinated according to the age-appropriate schedule showed higher pneumonia rates than vaccinated cases.

### 4.9. Cluster Analysis

Twenty-three cases (5.1% of the total cohort) were linked to identified household clusters. Six distinct family clusters were identified across three counties: three in Buzau county (including a cluster of seven cases in a single household, the largest single cluster identified), two in Constanta county, and one in Galati county. All clusters were family household in nature; no school-based, kindergarten-based, or healthcare facility clusters were identified. The confirmation rate in cluster cases was 82.6% (19 of 23) compared with 71.6% (307 of 429) in sporadic cases; this difference did not reach statistical significance (chi-square = 0.83, *p* = 0.362). Age distribution did not differ significantly between cluster and sporadic cases (Mann–Whitney U = 5138, *p* = 0.735; median age 3 years in both groups).

Cluster identification relied on the epidemiological-link field of the passive surveillance form, through which an investigating epidemiologist designated a case as outbreak-related and recorded the associated household or collectivity. No active contact-tracing or systematic household testing was undertaken. Consistent with the limitations of this passive approach, the probable site of transmission was recorded as unknown in 420 of 452 cases (92.9%), and although a median of 3 contacts per case was documented, with 264 cases (58.4%) reporting at least one household contact under 18 years of age, only 23 cases were formally linked to identified clusters. This discrepancy indicates that the low number of documented clusters most likely reflects under-ascertainment of household transmission inherent to passive surveillance rather than a true absence of intrafamilial spread, and the household cluster count reported here should be regarded as a minimum estimate.

### 4.10. Surveillance Quality Indicators

The median reporting delay across the full cohort was 14 days (IQR 8 to 20; range 0 to 64 days). Of 443 cases with valid delay data (interval 0 to 90 days), 392 (88.5%) were notified more than five days after symptom onset, exceeding the mandatory reporting deadline established under HG 657/2022 [31] by more than one week for the majority of the cohort.

To distinguish the contributions of patient-related and system-related processes to this interval, the reporting pathway was disaggregated into its component stages. The median interval from symptom onset to specimen collection was 11 days (IQR 6 to 15), whereas the interval from specimen collection to DSP notification was negligible (median 0 days, IQR 0 to 2), as was the interval from notification to initiation of epidemiological investigation (median 0 days). The pre-notification stage therefore accounted for almost the entire reporting delay, indicating that the observed interval predominantly reflects the timing of healthcare-seeking and clinical recognition of pertussis, which is rarely suspected before the paroxysmal stage begins one to two weeks after onset, rather than administrative inefficiency within the surveillance system, which responded promptly once cases entered it.

**Figure 10:**
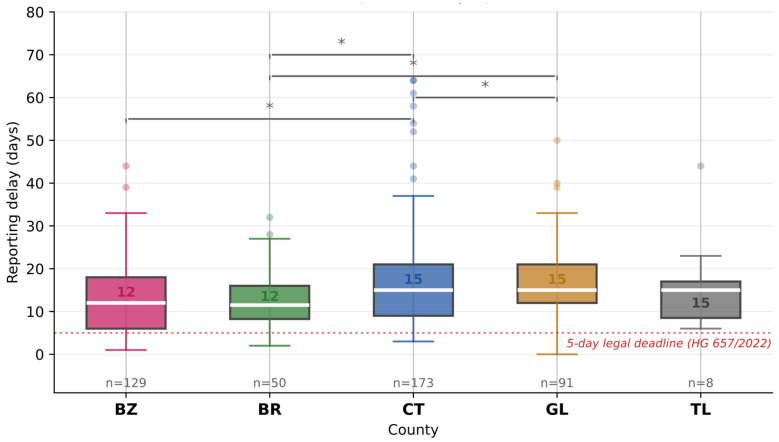
Reporting delay from symptom onset to DSP notification by county, with Kruskal–Wallis and post-hoc Bonferroni significance brackets, SE Romania, 2024.

Kruskal–Wallis analysis revealed significant inter-county differences in reporting delay (H = 21.85, df = 4, *p* = 0.0002; Figure 10). Median delays were shortest in Buzau and Braila (12 days, IQR 6 to 18 and IQR 8 to 16, respectively) and longest in Constanta and Galati (both 15 days, IQR 9 to 21 and IQR 12 to 21, respectively). Post-hoc pairwise Mann–Whitney tests with Bonferroni correction (adjusted alpha = 0.0083) identified four statistically significant county pairs: Buzau versus Constanta (*p* = 0.0004), Buzau versus Galati (*p* = 0.0007), Braila versus Galati (*p* = 0.0009), and Constanta versus Braila (*p* = 0.0064). No significant difference was observed between Buzau and Braila (*p* = 0.824) or between Constanta and Galati (*p* = 0.635).

Reporting delay showed a weak but statistically significant positive correlation with patient age (Spearman rho = 0.281, *p* below 0.001), indicating that cases in younger patients tended to be notified more promptly. A positive correlation was also identified between number of prior vaccine doses and reporting delay in confirmed cases (Spearman rho = 0.137, *p* = 0.013), consistent with delayed clinical recognition of attenuated presentations in partially vaccinated patients.

## 5. Discussion

### 5.1. Epidemiological Dynamics in the European Context

The pertussis epidemic documented in southeastern Romania during 2024 occurred in temporal and epidemiological synchrony with the broader EU/EEA resurgence described in the Introduction, confirming the global rather than locally contained nature of this post-pandemic phenomenon. The epidemic peak in September 2024, preceded by a progressive build-up from February onwards, reflects the late-summer to early-autumn seasonality characteristic of pertussis epidemics in temperate climates, where the return of children to school settings after summer recess amplifies respiratory pathogen transmission within communities already carrying a high susceptible burden. This seasonal pattern is consistent with observations from contemporaneous European outbreaks. In England, a national pertussis outbreak during 2023 to 2024 resulted in 15,750 laboratory-confirmed cases, with 12 infant deaths reported among 481 infants under three months of age, nine of whom were born to mothers not vaccinated within the recommended antenatal window [41]. The Danish epidemic, which began in August 2023, documented the highest reported case counts since systematic pertussis surveillance was established, with the most severe outcomes concentrated in infants below two months of age [42]. In Italy, a pertussis outbreak affecting neonates and young infants between January and May 2024 resulted in 108 hospitalisations and three deaths across 11 major paediatric centres, directly linked to non-compliance with antenatal immunisation recommendations [43]. The French resurgence during the same period was characterised by 5616 PCR-positive cases detected between January and May 2024 alone, following three years of near-zero incidence, with genomic analysis confirming the re-emergence of pertactin-expressing isolates and identification of a single macrolide-resistant strain [44]. Parallel resurgence was simultaneously documented in the United States, where pertussis case counts returned to pre-pandemic levels by 2024 [45]. Together, these observations confirm that the Romanian epidemic was part of a geographically widespread resurgence driven by common post-pandemic immunological mechanisms rather than country-specific factors, while the local features documented in the present study reflect Romania-specific vulnerabilities in vaccination coverage and surveillance infrastructure.

### 5.2. Vaccination Gaps as the Primary Modifiable Driver

The finding that 72.1% of confirmed cases were not vaccinated according to the age-appropriate national schedule represents the central public health message of this study. This proportion substantially exceeds the national DTP3 non-coverage estimate of approximately 15% derived from WHO/UNICEF 2022 data [6], demonstrating that vaccination gaps are not uniformly distributed across the population but are concentrated in symptomatic pertussis cases, consistent with well-established vaccine effectiveness data for the aP formulations in current use. The Netherlands, which recorded 18,208 pertussis notifications in 2024 (102 per 100,000 population), documented that 83% of mothers of infants notified under two months of age had not received maternal Tdap vaccination, and estimated maternal vaccination effectiveness against infant pertussis in the same period at 91% [46]. The protective effect of vaccination documented in Model B of the present study (adjusted OR = 0.53 for pneumonia in vaccinated versus unvaccinated confirmed cases, *p* = 0.035) is consistent with this evidence base and extends it to the Romanian epidemiological context by demonstrating statistically significant protection specifically against the most common severe complication of pertussis.

In contrast to the predominantly unvaccinated case profile in Romania, data from eastern China documented that 79.24% of pertussis cases confirmed in 2024 had received full vaccination according to schedule, with the interval between the last vaccine dose and illness onset having a median of 61.45 months, highlighting that waning immunity rather than non-vaccination constitutes the primary driver in high-coverage populations with sustained aP programmes [47]. This contrast is reinforced by our own data: although the interval since last vaccination increased markedly with age in our cohort, reaching a median of over 100 months in cases aged 10 years or above, this waning did not translate into severe outcomes, as pneumonia remained concentrated in the youngest unvaccinated infants. In this population, waning post-vaccination immunity contributed to transmission among older age groups without producing the severe clinical burden seen in early infancy. The coexistence of these two distinct epidemiological profiles globally underscores that pertussis resurgence is multifactorial: in Romania, primary prevention gaps (non-vaccination) predominate, whereas in settings with near-universal coverage, secondary prevention gaps (waning immunity and absence of booster programmes) drive resurgence. This distinction has direct policy implications, as the interventions required differ fundamentally between the two contexts.

The distribution of reasons for non-vaccination identified in the present study (parental refusal 43.0%, non-attendance 36.6%, combined 79.6%) confirms that the dominant barriers in this population are behavioural rather than structural or medical. Contraindications accounted for fewer than 0.5% of cases. This profile is consistent with national data on vaccine hesitancy trends in Romania and designates targeted communication and active recall interventions as the highest-yield public health strategies for improving DTPa coverage. The absence of a national maternal Tdap vaccination programme represents a structural gap that is particularly consequential given the high pneumonia rates documented in infants under two months of age in this study (81.0%), a group whose protection depends entirely on maternally derived passive immunity before the primary series can be initiated.

### 5.3. Age-Specific Vulnerability and Clinical Severity

The age distribution of confirmed cases, with 63.2% of patients under five years of age and a secondary component of adult cases (7.1% aged 18 years or above), reflects the dual-peak demographic pattern increasingly characterising post-pandemic pertussis epidemics across Europe [4] and is consistent with the interplay between accumulation of unvaccinated young children and waned immunity in older age groups. The independent protective effect of vaccination against pneumonia (adjusted OR = 0.53, 95% CI 0.29 to 0.96, *p* = 0.035) in Model B, maintained after adjustment for age group, sex, and residential setting, provides direct multivariate evidence that on-schedule DTPa vaccination reduces the risk of the most frequent severe complication of pertussis beyond its established effect on overall case incidence. The strong inverse gradient in pneumonia rates with increasing age (chi-square = 49.46, *p* below 0.001) and the odds ratio of 4.13 for pneumonia in infants under one year compared with older patients confirm that the youngest age group bears a disproportionate burden of severe outcomes, a finding consistent with the pathobiological mechanisms of pertussis-associated respiratory compromise in immature airways.

The borderline association between rural residential setting and higher pneumonia risk (adjusted OR = 1.64, *p* = 0.055) in Model B did not reach the pre-specified significance threshold but is biologically plausible and consistent with documented disparities in emergency healthcare access and vaccination coverage between urban and rural areas in Romania. This association warrants confirmation in larger prospective studies. The importance of comprehensive and multidimensional health monitoring in paediatric and adolescent populations, integrating clinical, biological, and quality-of-life dimensions, has been highlighted in complementary institutional research using validated instruments including the SF-36 and PHQ-9 [48] and in studies of corrective health interventions for postural disorders in children and adolescents [49]. Beyond the direct clinical burden, the impact of acute infectious illness on the family unit and the quality of life of caregivers represents an additional dimension of pertussis-associated morbidity that warrants consideration in comprehensive disease burden assessments [50].

### 5.4. Surveillance System Performance and Data Quality

The systematic inconsistency between original case classifications and laboratory results in the surveillance database, with 97.6% of PCR-positive cases originally classified as infirmed, represents a data quality finding of direct relevance to the interpretation of the epidemiological results and to the design of future surveillance systems. This pattern is most consistent with classifications having been finalised at the time of initial clinical notification, prior to receipt of laboratory results, without a mechanism for subsequent mandatory record update. An analogous discrepancy between surveillance database classifications and definitive laboratory confirmation has been documented in passive pertussis surveillance systems across multiple EU/EEA countries and identified as a recognised data quality challenge [7]. The pre-specified reclassification rule applied in the present study addresses this limitation transparently; however, the underlying systemic deficiency in the national EpiInfo workflow requires resolution through the introduction of mandatory classification review triggers at the point of laboratory result receipt.

The 88.5% rate of cases notified more than five days after symptom onset, systematically exceeding the mandatory five-day reporting deadline established under HG 657/2022 [31], reflects an inherent tension between the legal notification requirement and the clinical reality of pertussis: the diagnosis is rarely suspected before the paroxysmal stage, which typically begins seven to fourteen days after symptom onset. This structural constraint explains why reporting delay cannot be reduced to below the statutory threshold in the majority of pertussis cases through administrative measures alone, and argues for a revision of reporting expectations in the national legislation to acknowledge the diagnostic timeline of the disease. Nonetheless, the significant inter-county variability in reporting delay (Kruskal–Wallis H = 21.85, *p* = 0.0002), with Buzau and Braila demonstrating shorter median delays than Constanta and Galati, and the positive correlation between delay and patient age (Spearman rho = 0.281, *p* below 0.001), suggest that DSP-level operational factors and clinical recognition patterns contribute independently to reporting timeliness beyond the biological constraints of disease progression.

This interpretation is directly supported by the stage-specific decomposition of the reporting pathway: the interval from specimen collection to notification and from notification to epidemiological investigation were both negligible (median 0 days), whereas the pre-notification interval from symptom onset to specimen collection accounted for almost the entire delay (median 11 days). Surveillance system performance after case entry was therefore prompt, and the observed delay is attributable predominantly to the pre-diagnostic phase of patient healthcare-seeking and clinical recognition rather than to administrative processing within the system.

The significant heterogeneity in PCR implementation rates across counties (chi-square = 93.67, *p* below 0.001), ranging from 72% in Buzau to 100% in Braila and Galati, has a direct and quantifiable consequence for surveillance data: the confirmation rate in Buzau (52.7%) is substantially lower than in Galati (90.1%) or Braila (96.0%), and this differential reflects diagnostic underascertainment rather than a true difference in pertussis burden. Policies equalising PCR access and implementation standards across county public health laboratories, including investment in rapid molecular diagnostic platforms and standardisation of specimen transport protocols, are essential prerequisites for achieving comparable surveillance quality and valid inter-county comparisons. A direct consequence of this near-complete reliance on PCR is the loss of capacity for antimicrobial susceptibility testing and strain characterisation, which require viable isolates; this limits the ability of the national system to monitor the emergence of macrolide-resistant *B. pertussis*, increasingly reported elsewhere in Europe, and represents a surveillance gap of growing public health relevance.

### 5.5. Public Health Implications

The findings of this study translate into four concrete public health priorities. First, restoring and sustaining childhood DTPa coverage above the 95% herd-immunity threshold requires targeted catch-up immunisation for children with incomplete or delayed schedules; given that 57.1% of confirmed cases had received no doses at illness onset, active identification and recall of un- and under-vaccinated children through the primary-care and school-health networks, supported by electronic immunisation registries to flag missed appointments, represents the highest-yield intervention.

Second, because parental refusal (43.0%) and non-attendance (36.6%) together accounted for almost 80% of non-vaccination, while medical contraindications were negligible (<0.5%), the dominant barriers are behavioural rather than structural. Evidence-based responses to vaccine hesitancy, including motivational-interview-based provider training, presumptive recommendation approaches, and locally tailored communication addressing specific community concerns, particularly in rural and under-served areas, should be prioritised over generic awareness campaigns.

Third, the high pneumonia burden in infants under two months of age (81.0%), who are too young for the first vaccine dose, underscores the urgency of introducing a national maternal Tdap programme. Implementation during the third trimester (27–36 weeks gestation), integrated into routine antenatal care with provider-level recommendation, is the most effective available strategy for protecting this group through transplacental antibody transfer and should be adopted as national policy.

Fourth, the surveillance deficiencies identified here, heterogeneous PCR access, predominantly clinical case confirmation, and inconsistent post-laboratory reclassification, call for specific system improvements: equalising molecular diagnostic capacity across county laboratories, introducing mandatory case-classification review triggered by laboratory result receipt, and strengthening active contact tracing to improve detection of household transmission.

### 5.6. Strengths

The present study offers several methodological strengths. It represents the first peer-reviewed epidemiological analysis of the 2024 pertussis resurgence at the subnational level in Romania, filling a documented gap in the national evidence base. Data were collected through a standardised national surveillance instrument applied uniformly across all five study counties, ensuring consistency in variable definitions and collection methods. The sample size of 326 confirmed cases supported multivariate logistic regression modelling with adequate statistical power for the pre-specified covariates. The pre-specified and transparently reported reclassification rule addresses a documented data quality limitation while maintaining methodological reproducibility.

### 5.7. Study Limitations: Several Limitations Require Acknowledgement

First, the passive surveillance design is inherently subject to under-notification bias, particularly for mild or atypical presentations in vaccinated adolescents and adults; the 7.1% adult confirmed fraction almost certainly underestimates the true adult burden. Second, the classification inconsistency in the database, although addressed through reclassification, may have introduced residual misclassification in a small number of cases. Third, an overall crude incidence rate was estimated using 2021 census denominators, the absence of official disaggregated 2024 resident-population figures at county and age-group level precluded calculation of county-specific and age-specific incidence rates; these will be incorporated when INS-INSSE data become available and represent a priority for future analysis. Fourth, the single-year scope of the dataset precludes analysis of temporal trends across epidemic cycles. Fifth, the absence of subnational DTP3 and booster coverage data at county level prevents direct ecological analysis of the coverage-incidence relationship. Sixth, the observational design and absence of a control group limit the causal interpretation of associations identified in multivariate models; the protective effect of vaccination against pneumonia, while statistically significant after adjustment, should be considered an adjusted association rather than a definitive causal estimate. Seventh, laboratory confirmation was available for only 27.3% of confirmed cases, the remainder being confirmed on clinical grounds in accordance with the national case definition; this reflects the predominantly clinical basis of confirmation in passive surveillance and may affect comparability with laboratory-based surveillance systems. Eighth, household transmission was likely under-ascertained, as cluster detection relied on passive epidemiological linkage without active contact tracing. Ninth, the national surveillance form does not capture data on underlying health conditions, prematurity, or other comorbidities that may influence disease severity; consequently, the analysis of severity determinants could not adjust for these factors, and the possibility of residual confounding by unmeasured underlying conditions cannot be excluded. Tenth, antimicrobial resistance surveillance was not performed: macrolide susceptibility testing requires a viable cultured isolate, and culture was positive in only a single case across the entire cohort, reflecting the near-complete reliance on PCR-based diagnosis. Systematic monitoring of macrolide resistance in circulating *B. pertussis* strains was therefore not feasible within this surveillance framework.

## 6. Conclusions

The present study documents a substantial pertussis resurgence in southeastern Romania during 2024, with 326 confirmed cases across five counties of Romania’s South-East development region. The epidemic peaked between August and October 2024, in temporal synchrony with the broader EU/EEA resurgence, and occurred in a context shaped by post-pandemic immunity debt, suboptimal vaccination coverage, and renewed social mixing.

Children under five years of age represented almost two thirds of confirmed cases, while infants were the group at highest clinical risk. Pneumonia was most frequent among the youngest infants, confirming that children too young to be fully immunised remain the priority target for preventive strategies. Vaccination gaps were the main modifiable determinant identified in this cohort: 72.1% of confirmed cases were not vaccinated according to the age-appropriate schedule, and most documented non-vaccination was attributable to parental refusal or non-attendance at scheduled appointments.

Age-appropriate vaccination was independently protective against pneumonia, supporting the clinical value of timely immunisation in reducing disease severity. These findings reinforce the need to restore childhood DTPa coverage above the 95% herd-immunity threshold through targeted communication, active recall systems, and improved follow-up of missed appointments. The introduction of a national maternal Tdap vaccination programme should also be prioritised, given the high vulnerability of infants below the age of first vaccine eligibility.

The study also identified inter-county differences in PCR implementation and reporting delays, together with inconsistencies between laboratory results and final case classifications. Harmonisation of laboratory access, mandatory post-laboratory classification review, and improved surveillance workflows are needed to strengthen the comparability and timeliness of pertussis surveillance data. Future analyses should incorporate official county-specific population denominators and subnational vaccination coverage data to complete incidence estimation and enable direct modelling of the coverage–incidence relationship.

## Figures and Tables

**Table 1:** General characteristics of the reported and confirmed pertussis cases, southeastern Romania, 2024.

Characteristic	Category	Total (n = 452)	Confirmed (n = 326)
Sex	Female, n (%)	245 (54.2%)	173 (53.1%)
	Male, n (%)	207 (45.8%)	153 (46.9%)
Residential setting	Urban, n (%)	263 (58.2%)	203 (62.3%)
	Rural, n (%)	189 (41.8%)	123 (37.7%)
Age	Mean years (SD) *	6.1 (9.7)	5.6 (9.0)
	Median years [IQR]	3 [0–7]	3 [0–6]
	Below 1 year, n (%)	127 (28.1%)	94 (28.8%)
	1 to 4 years, n (%)	159 (35.2%)	112 (34.4%)
	5 to 9 years, n (%)	84 (18.6%)	62 (19.0%)
	10 to 14 years, n (%)	39 (8.6%)	29 (8.9%)
	15 to 17 years, n (%)	9 (2.0%)	6 (1.8%)
	18 years or above, n (%)	34 (7.5%)	23 (7.1%)
Vaccination status	Vaccinated per age, n (%)	110 (24.3%)	91 (27.9%)
	Not vaccinated per age, n (%)	342 (75.7%)	235 (72.1%)
	0 doses received, n (%)	280 (61.9%)	186 (57.1%)
	1 to 2 doses, n (%)	83 (18.4%)	48 (14.7%)
	3 doses or above, n (%)	89 (19.7%)	92 (28.2%)
Laboratory investigation	PCR performed, n (%)	354 (78.3%)	290 (88.9%)
	PCR positive, n (%)	83 (18.4%)	82 (25.2%)
	Serology performed, n (%)	210 (46.5%)	50 (15.3%)
Reporting delay	Median days [IQR **]	14 [8–20]	13 [7–19]
	Reported within 5 days, n (%)	52 (11.5%)	39 (12.0%)
Complications (confirmed only)			120 (36.8%)
			2 (0.6%)
			1 (0.3%)
			0 (0%)

* SD = standard deviation; ** IQR = interquartile range.

**Table 2:** County-level distribution, confirmation rates, PCR testing rates, and reporting delays among reported pertussis cases.

County	Total n (%)	Confirmed n (%)	Confirmation Rate	PCR Rate * (Confirmed)	Median Delay Days [IQR]
Braila (BR)	50 (11.1%)	48 (96.0%)	96.0%	100%	12 [8–16]
Buzau (BZ)	129 (28.6%)	68 (52.7%)	52.7%	72%	12 [6–18]
Constanta (CT)	173 (38.4%)	122 (70.5%)	70.5%	87%	15 [9–21]
Galati (GL)	91 (20.2%)	82 (90.1%)	90.1%	99%	15 [12–21]
Tulcea (TL)	8 (1.8%)	6 (75.0%)	75.0%	100%	15 [8–17]
**Total**	**451 (100%)**	**326 (72.3%)**		**89%**	**14 [8–20]**

* Chi-square (county vs. confirmed, post-reclassification) = 53.43, df = 4, *p* below 0.001. Chi-square (PCR rate by county, all reported cases) = 93.67, df = 4, *p* below 0.001. IQR = interquartile range.

**Table 3:** Pneumonia frequency and vaccination-dose distribution by age group among confirmed pertussis cases.

Age Group	Confirmed (n)	Pneumonia n (%)	Vaccinated Per Age n (%)	0 Doses n (%)	1–2 Doses n (%)	3 Doses or Above n (%)
Below 2 months	21	17 (81.0%)	1 (4.8%)	21 (100%)	0	0
2 to 5 months	48	24 (50.0%)	9 (18.8%)	35 (72.9%)	13 (27.1%)	0
6 to 11 months	25	16 (64.0%)	4 (16.0%)	19 (76.0%)	6 (24.0%)	0
1 to 4 years	112	41 (36.6%)	32 (28.6%)	64 (57.1%)	15 (13.4%)	33 (29.5%)
5 to 9 years	62	15 (24.2%)	21 (33.9%)	23 (37.1%)	11 (17.7%)	28 (45.2%)
10 to 14 years	29	5 (17.2%)	15 (51.7%)	6 (20.7%)	3 (10.3%)	20 (69.0%)
15 to 17 years	6	0 (0%)	4 (66.7%)	0	0	6 (100.0%)
18 years or above	23	2 (8.7%)	N/A *	18 (78.3%)	0	5 (21.7%)
**Total**	**326**	**120 (36.8%)**	**91 (27.9%)**	**186 (57.1%)**	**48 (14.7%)**	**92 (28.2%)**

* N/A indicates that vaccination per age-appropriate schedule is not applicable as a binary measure for adults beyond the primary schedule.

**Table 4:** Multivariable logistic regression models for confirmed diagnosis and pneumonia.

Predictor	Model A: Confirmed Diagnosis			Model B: Pneumonia (Confirmed)		
	OR (adj.) *	95% CI	*p*	OR (adj.)	95% CI	*p*
Male sex (ref: female)	0.92	0.60–1.40	0.692	0.64	0.39–1.05	0.078
Rural residence (ref: urban)	**0.56**	**0.37–0.86**	**0.008**	1.64	0.99–2.72	0.055
Vaccinated per age (ref: not vaccinated)	**2.16**	**1.24–3.76**	**0.006**	**0.53**	**0.29–0.96**	**0.035**
Age group (ordinal, per category)	0.91	0.76–1.08	0.276	**0.51**	**0.40–0.65**	**<0.001**
Model summary	n = 452; omnibus χ^2^ = 16.95; *p* = 0.002; Nagelkerke R^2^ = 0.053			n = 326; omnibus χ^2^ = 54.95; *p* < 0.001; Nagelkerke R^2^ = 0.212; Hosmer–Lemeshow *p* = 0.352		

* OR adj. = adjusted odds ratio; CI = confidence interval. Bold values indicate *p* < 0.05. Age group ordinal categories: 0, below 1 year; 1, 1 to 4 years; 2, 5 to 9 years; 3, 10 to 17 years; 4, 18 years or above. Models built using the enter method in IBM SPSS Statistics version 29.0. In the primary models, the full analytical cohorts were retained. Because age-appropriate vaccination status is not directly applicable to adults in the Romanian schedule, sensitivity analyses excluding adults aged 18 years or above were performed and are reported in the text.

## Data Availability

The data presented in this study are not publicly available, as they were obtained as part of national communicable disease surveillance and provided by the National Institute of Public Health (INSP-CNSCBT) upon official request, for use with attribution to the source of provenance.

## Abbreviations

ACT/CyaA	Adenylate cyclase toxin
ADP	Adenosine diphosphate
AI	Artificial intelligence
aP	Acellular pertussis (vaccine)
BR	Braila (county)
BvgAS	Bordetella virulence gene two-component regulatory system
BZ	Buzau (county)
cAMP	Cyclic adenosine monophosphate
CD4+	Cluster of differentiation 4 positive (T cells)
CDC	Centers for Disease Control and Prevention
CI	Confidence interval
CNSCBT	National Centre for Surveillance and Control of Communicable Diseases
COVID-19	Coronavirus disease 2019
CRSP	Regional Public Health Centre (Centrul Regional de Sanatate Publica)
CT	Constanta (county)
DALY	Disability-adjusted life year
df	Degrees of freedom
DSP	County Public Health Directorate (Directia de Sanatate Publica)
DTaP	Diphtheria, tetanus, acellular pertussis (vaccine)
DTP3	Third dose of diphtheria-tetanus-pertussis vaccine
DTwP	Diphtheria, tetanus, whole-cell pertussis (vaccine)
ECDC	European Centre for Disease Prevention and Control
EEA	European Economic Area
ELISA	Enzyme-linked immunosorbent assay
EU	European Union
FHA	Filamentous hemagglutinin
GBD	Global Burden of Disease
GDPR	General Data Protection Regulation
GL	Galati (county)
HepB	Hepatitis B
HG	Government Decision (Hotarare de Guvern)
Hib	Haemophilus influenzae type b
IgG	Immunoglobulin G
INS	National Institute of Statistics (Institutul National de Statistica)
INSP	National Institute of Public Health (Institutul National de Sanatate Publica)
IPV	Inactivated poliovirus vaccine
IQR	Interquartile range
IS481/IS1001	Bordetella pertussis insertion sequences
IU/mL	International units per millilitre
ML	Machine learning
NUTS	Nomenclature of Territorial Units for Statistics
OR	Odds ratio
PAR	Population attributable risk
PCR	Polymerase chain reaction
PHQ-9	Patient Health Questionnaire-9
PRN	Pertactin
PT	Pertussis toxin
qPCR	Quantitative polymerase chain reaction
RR	Relative risk
SD	Standard deviation
SF-36	36-Item Short Form Health Survey
SPSS	Statistical Package for the Social Sciences
Tdap	Tetanus, diphtheria, acellular pertussis (vaccine)
TESSy	The European Surveillance System
Th1/Th2/Th17	T helper cell type 1/2/17
TL	Tulcea (county)
UNICEF	United Nations Children’s Fund
VE	Vaccine effectiveness
WHO	World Health Organization
wP	Whole-cell pertussis (vaccine)
